# Supplementary material for: Asian summer monsoon variability across Termination II and implications for ice age terminations
Source: Nat Commun. 2025 May 30;16:5025. doi: 10.1038/s41467-025-60398-w (PMC12125170; doi:10.1038/s41467-025-60398-w)
Supplement: Supplementary file 1 — Supplementary Information [file 41467_2025_60398_MOESM1_ESM.pdf]

Supplementary Information for

Asian summer monsoon variability across Termination II and implications for ice age terminations

Yijia Liang[1,2], Kan Zhao[1]*, Yongjin Wang[1]*, Shitao Chen[1], Tyler E. Huth[3,4], Bin Zhao[1], Quan Wang[5], Zhenqiu Zhang[1], Qingfeng Shao[1], Hai Cheng[6], R. Lawrence Edwards[7]

1 State Key Laboratory of Climate System Prediction and Risk Management, Jiangsu Center for Collaborative Innovation in Geographical Information Resource Development and Application, School of Geography, Nanjing Normal University, Nanjing 210023, China

2 School of Geographical Science, Nantong University, Nantong 226007, China

3 Department of Earth, Environmental, and Planetary Sciences, Washington University, St. Louis 63130, USA

4 Department of Earth and Environmental Sciences, University of Michigan, Ann Arbor 48109, USA

5 Research Centre for Environmental Change and Sustainable Development, School of International Business and Tourism Management, Ningbo Polytechnic, Ningbo 315800, China

6 Institute of Global Environmental Change, Xi'an Jiaotong University, Xi'an 710049, China

7 Department of Earth and Environmental Sciences, University of Minnesota, Minneapolis 55455, USA

*Corresponding authors. Email: 09371@njnu.edu.cn (Z.K.), yjwang@njnu.edu.cn (W.Y.)

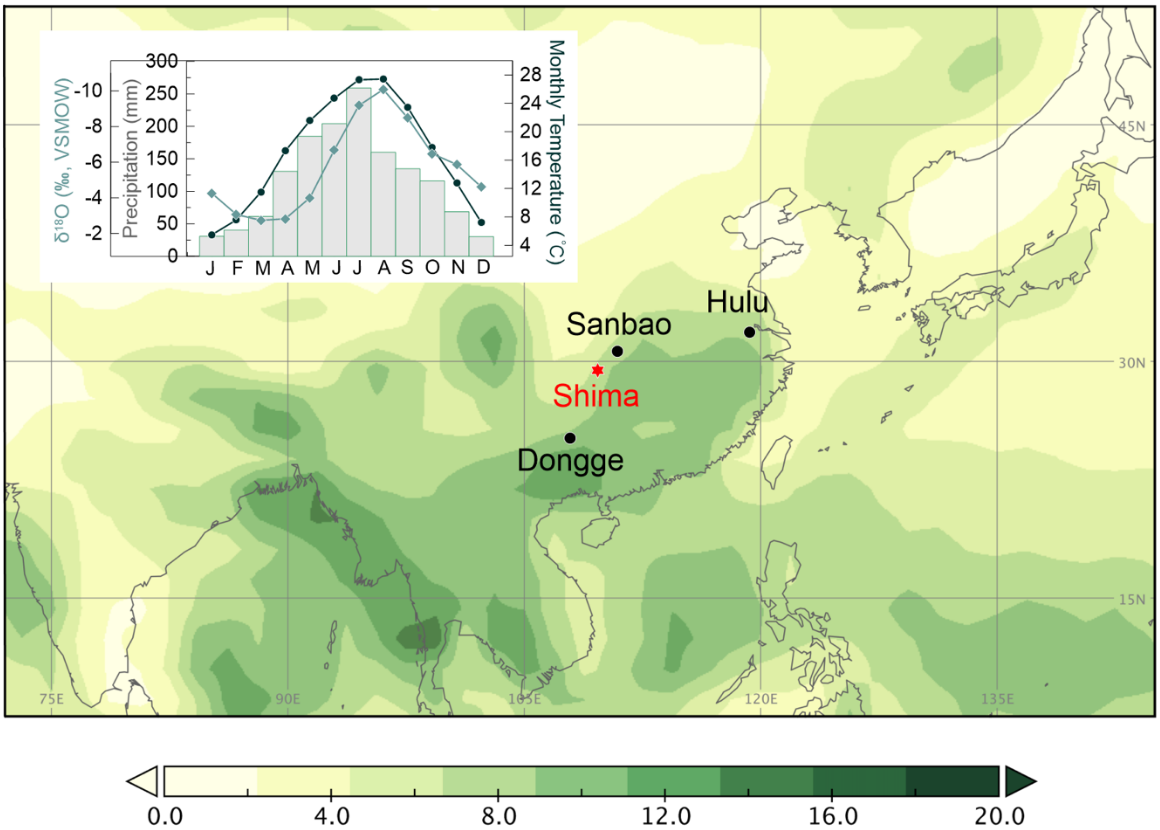

Fig. 1 Regional climatology and cave sites. Base map is the NCEP/NCAR May to August (MJJA) daily precipitation (mm/day) averaged from 1983 to 2012, reproduced by Panoply Data Viewer (https://www.giss.nasa.gov/tools/panoply/download/) with data from (https://psl.noaa.gov/cgi-bin/data/composites/printpage.pl). Red star denotes the location of Shima Cave. Black dots are locations of Hulu Cave[1,2], Sanbao Cave[3], and Dongge Cave[4]. Inset shows monthly rainfall (bar chart) and temperature (dark green points) at Enshi station (30°16′N, 109°28′E, 80 km distant from Shima Cave, data sourced from https://gis.ncdc.noaa.gov/maps/ncei/cdo/monthly), and monthly simulated precipitation $\delta^{18}O$[5] (light green diamonds).

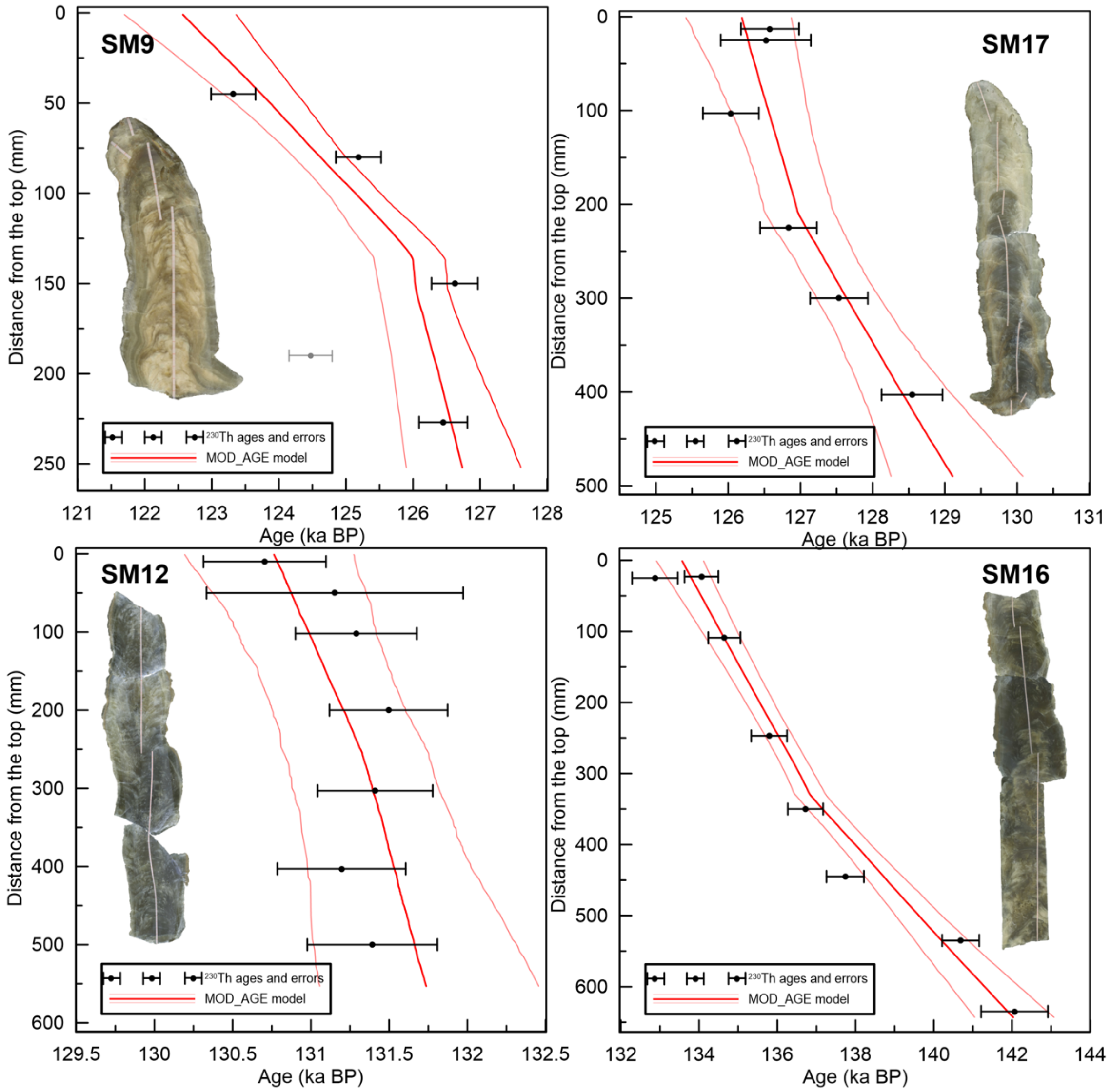

Fig. 2 Polished profiles and age models produced using the MOD_AGE model[6] for four samples from Shima Cave. Black dots and error bars indicate $^{230}Th/U$ ages and 2σ dating errors. Thick and thin red lines represent the age median and 2σ results. An outlier of sample SM9 is marked grey, and all the rest are fairly within the modeled errors. Source data are provided as a Source Data file, the same as in Figure 1a.

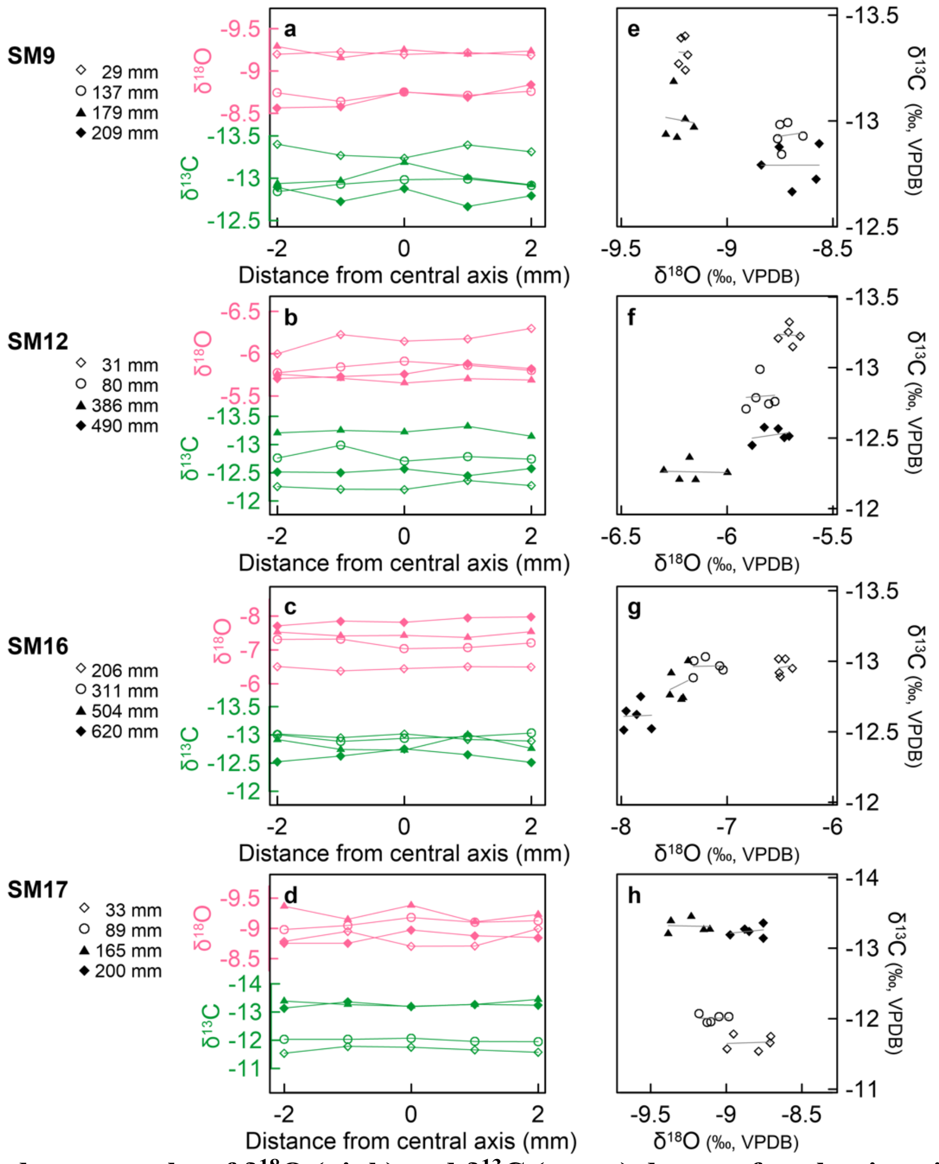

Fig. 3 Hendy test results of $\delta^{18}O$ (pink) and $\delta^{13}C$ (green) data on four laminae in each stalagmite. a-d Results for individual layers. **e-h** Plots of $\delta^{13}C$ and $\delta^{18}O$ with linear fit. Source data are provided as a Source Data file, the same as in Figure 1b.

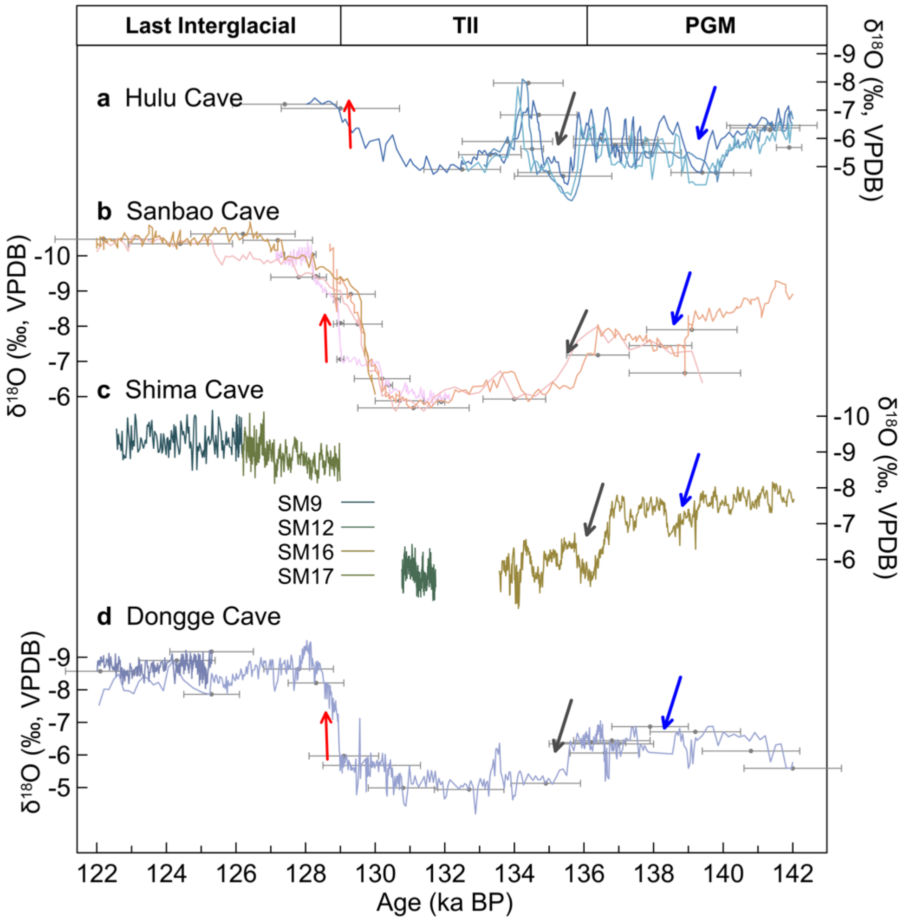

Fig. 4 Comparison of Chinese stalagmite δ^{18}O records. Records are from **a** Hulu Cave[1,2], **b** Sanbao Cave[3,7], **c** Shima Cave (this study) and **d** Dongge Cave[4], with dots and error bars indicating ^{230}Th dating and 2σ errors. Arrows indicate the abrupt shifts at around 129 ka BP (red arrows), 136 ka BP (black arrows), and the ~139-ka weak monsoon intervals (blue arrows) consistent across different cave records. All records are on their independent age models. TII=Termination II, PGM=Penultimate Glacial Maximum. Source data are provided as a Source Data file.

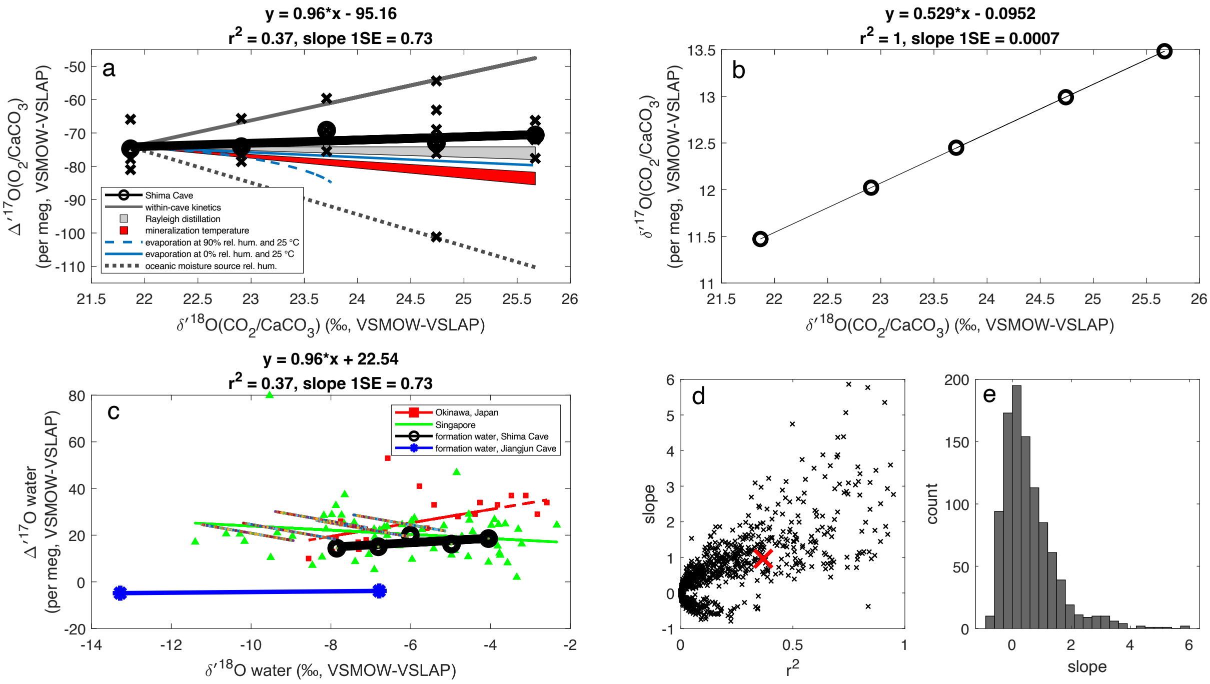

Fig. 5 Triple oxygen isotope data for Shima Cave sample SM7. a Shima Cave Δ′^{17}O vs. δ′^{18}O data (averages: black dots, replicates: black crosses) and trend (bold black line) compared to characteristic trends for hydrologic processes: within-cave kinetics (solid dark gray), Rayleigh distillation at 25 °C (medium gray polygon), pan evaporation endmembers (solid and dashed blue), mineralization temperature (red polygon), and oceanic moisture source relative humidity (black dotted) (rel. hum. = relative humidity). **b** Comparison of δ′^{17}O and δ′^{18}O data with best fit line. **c** Modern and inferred formation water isotope composition: Okinawa, Japan[8] (red squares), Singapore[9] (green triangles), Shima Cave (black circles; this study), and Jiangjun Cave, China[10] (blue circles). Shima Cave values are shown for the modern formation temperature of 17 °C. A Monte Carlo sensitivity test (n = 1000) for Shima Cave formation temperatures 5–15 °C cooler than modern is shown in the strings of colored dots. **d** Slope vs. r^2 for the best fit line of individual simulations, with red cross marking the 17 °C scenario (i.e., for the black circles in **c**). **e** Histogram of the slopes of best fit lines for the Monte Carlo sensitivity test. Source data and Matlab Code are provided as a Source Data file.

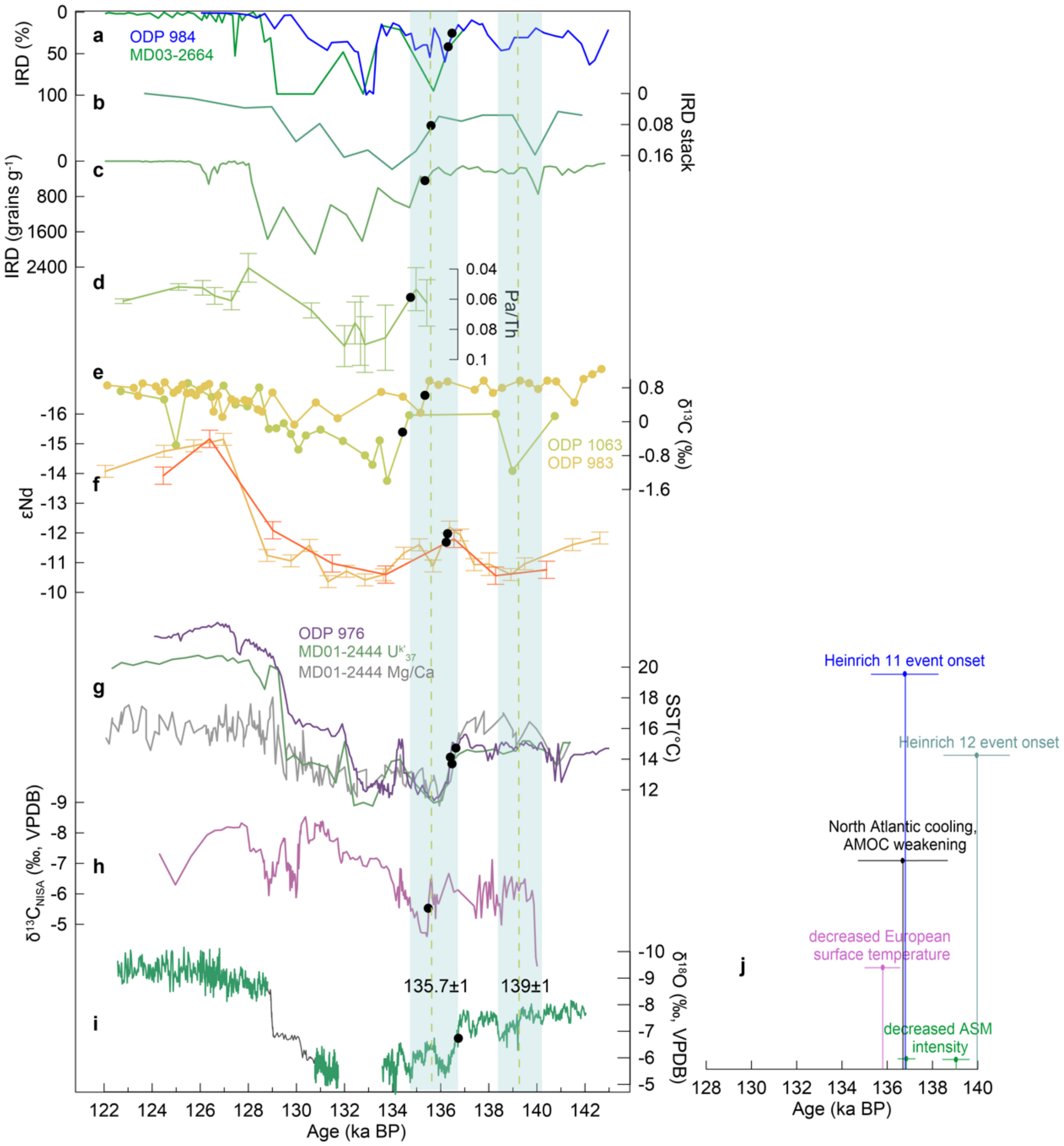

Fig. 6 Timing of abrupt shifts in different oceanic and terrestrial records. Ice rafted debris (IRD) proxies in the North Atlantic: **a** IRD percentages from cores ODP 984[11,12] and MD03-2664[13], **b** compiled IRD stack[14], and **c** IRD record from core ODP 983[15]. Atlantic Meridional Overturning Circulation (AMOC) proxies: **d** Pa/Th record from core ODP 1063[16], **e** benthic $\delta^{13}C$ records from cores ODP 983 and ODP 1063[17] and **f** εNd records from ODP 1063[16,17]. Sea surface temperature (SST) proxies in the North Atlantic: **g** proxy records from cores ODP 976 and MD01-2444[12,18]. European surface temperature: **h** $\delta^{13}C_{NISA}$ record from NW Iberian caves[19]. Asian summer monsoon (ASM) intensity proxy: **i** $\delta^{18}O$ records from Sanbao Cave[7] (black) and Shima Cave (green, this study). Black dots indicate the timing of abrupt changes in different records. Green dashed lines and green bars indicate medium ages and errors for two important abrupt shifts of the 139-ka event and the onset of Termination II. **j** timing of changes in climatic elements from different records with age error bars. We applied the smallest age errors with the best age constraints provided by the references. Source data are provided as a Source Data file.

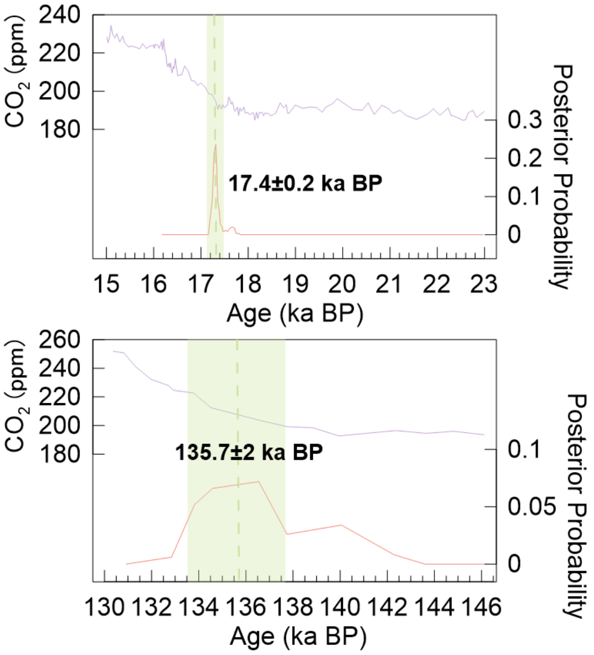

Fig. 7 Change points in EDC CO_2 record[20] during Termination I and Termination II. Data were obtained using Change Point detector in software Acycle 2.8[21]. The median ages of 17.4 and 135.7 ka BP are the peak center values (dashed lines) in posterior probability and the errors of ±200 and ±2000 years (shaded area) bracket the major body of the peak. The Acycle 2.8 software[21] can be found at https://acycle.org. Source data are provided as a Source Data file.

Table 1 ^{230}Th dating results of stalagmite samples from Shima Cave

Sample Number	^{238}U (ppb)		^{232}Th (ppt)		^{230}Th / ^{232}Th (atomic x10^{-6})		$\delta^{234}U$* (measured)		^{230}Th / ^{238}U (activity)		^{230}Th Age (ka) (uncorrected)		$\delta^{234}U_{Initial}$** (corrected)		^{230}Th Age (ka BP)*** (corrected)	
SM12-10	94.6	±0.1	19.2	±1	99796	±6239	652	±2	1.2298	±0.0017	130.78	±0.39	943	±3	130.70	±0.39
SM12-50N	171.5	±0.3	114.5	±4	30624	±1012	662	±3	1.2401	±0.0038	131.23	±0.82	958	±5	131.15	±0.82
SM12-102	176.7	±0.2	178.1	±4	20207	±443	655	±1	1.2358	±0.0018	131.37	±0.39	950	±2	131.29	±0.39
SM12-200	197.4	±0.2	233.0	±5	18236	±387	739	±2	1.3056	±0.0018	131.58	±0.38	1072	±2	131.50	±0.38
SM12-303	238.0	±0.2	153.4	±3	33697	±759	754	±2	1.3172	±0.0016	131.49	±0.37	1093	±3	131.41	±0.37
SM12-403	219.3	±0.2	191.0	±4	24579	±536	732	±2	1.2982	±0.0018	131.28	±0.41	1061	±3	131.20	±0.41
SM12-500	200.6	±0.2	82.4	±2	54048	±1381	789	±2	1.3458	±0.0019	131.47	±0.42	1144	±3	131.39	±0.42
SM16-23	347.8	±0.3	198.0	±5	40292	±946	829	±2	1.3941	±0.0021	134.14	±0.43	1210	±3	134.06	±0.43
SM16-25N	344.6	±0.4	381.6	±9	20676	±500	831	±2	1.3886	±0.0032	132.96	±0.58	1209	±3	132.87	±0.58
SM16-109	373.8	±0.4	248.0	±5	33952	±752	794	±2	1.3682	±0.0020	134.73	±0.41	1160	±3	134.65	±0.41
SM16-247	291.5	±0.4	95.0	±3	70283	±1921	809	±2	1.3877	±0.0021	135.87	±0.46	1187	±3	135.79	±0.46
SM16-350	475.6	±0.5	1315.0	±26	8212	±166	789	±2	1.3766	±0.0021	136.83	±0.45	1161	±3	136.72	±0.45
SM16-445	430.3	±0.5	4812.0	±96	2077	±42	820	±2	1.4086	±0.0023	137.96	±0.47	1209	±3	137.74	±0.48
SM16-535	395.2	±0.4	432.0	±9	21395	±441	812	±2	1.4178	±0.0022	140.77	±0.47	1208	±3	140.69	±0.47
SM16-635N	367.6	±0.5	256.7	±7	33787	±938	818	±2	1.4307	±0.0045	142.14	±0.85	1222	±4	142.07	±0.85
SM17-13	489.3	±0.5	561.7	±12	15214	±316	466	±2	1.0593	±0.0014	126.67	±0.40	667	±3	126.58	±0.40
SM17-25N	471.1	±0.6	429.0	±10	19067	±442	459	±1	1.0531	±0.0028	126.61	±0.62	656	±2	126.52	±0.62
SM17-103	563.8	±0.6	265.2	±6	38801	±885	530	±2	1.1069	±0.0014	126.11	±0.39	756	±3	126.04	±0.39
SM17-225	471.1	±0.5	123.3	±3	72822	±1981	586	±2	1.1559	±0.0015	126.91	±0.39	838	±3	126.83	±0.39
SM17-300	589.1	±0.6	631.9	±13	17984	±367	599	±2	1.1699	±0.0015	127.62	±0.40	858	±3	127.53	±0.40
SM17-403	495.4	±0.5	6209.8	±124	1560	±31	611	±2	1.1861	±0.0015	128.81	±0.40	878	±3	128.55	±0.42
SM9-45N	453.2	±0.2	857.7	±18	8159	±174	329	±1	0.9360	±0.0012	123.36	0.33	466	±1	123.32	±0.33
SM9-80N	317.6	±0.1	738.0	±7	6098	±59	222	±1	0.8590	±0.0010	125.24	0.34	316	±1	125.19	±0.34
SM9-150N	304.8	±0.1	414.8	±6	10203	±148	194	±1	0.8419	±0.0011	126.65	0.34	277	±1	126.62	±0.34
SM9-190N	367.7	±0.1	497.9	±6	10574	±134	237	±1	0.8681	±0.0011	124.50	0.32	337	±1	124.47	±0.32
SM9-227N	299.1	±0.1	632.8	±14	6883	±150	246	±1	0.8829	±0.0012	126.50	0.36	351	±1	126.45	±0.36

* $\delta^{234}U = ([^{234}U/^{238}U]_{activity} - 1) \times 1000$.
** $\delta^{234}U_{initial}$ was calculated based on ^{230}Th age (T), i.e. $\delta^{234}U_{initial} = \delta^{234}U_{measured} \times e^{\lambda 234 x T}$. U decay constants: $\lambda_{238} = 1.55125 \times 10^{-10}$ yr^{-1} ref.[22] and $\lambda_{234} = 2.82206 \times 10^{-6}$ yr^{-1} ref.[23]. Th decay constant: $\lambda_{230} = 9.1705 \times 10^{-6}$ yr^{-1} ref.[23].
*** Corrected ^{230}Th ages assume the initial $^{230}Th/^{232}Th$ atomic ratio of $(4.4 \pm 2.2) \times 10^{-6}$. Those are the values for a material at secular equilibrium, with the bulk earth $^{232}Th/^{238}U$ value of 3.8. Errors are 2σ analytical errors. All ages are corrected to 'present' which is defined as the year 1950 AD.
Most of the dating samples were measured at the University of Minnesota and nine subsamples with "N" character were measured at Nanjing Normal University.

References

1. Cheng, H. *et al.* A penultimate glacial monsoon record from Hulu Cave and two-phase glacial terminations. *Geology* **34**, 217 (2006).
2. Wang, Q. *et al.* Millennial-scale Asian monsoon variability during the late Marine Isotope Stage 6 from Hulu Cave, China. *Quat. Res.* **90**, 394–405 (2018).
3. Wang, Y. *et al.* Millennial- and orbital-scale changes in the East Asian monsoon over the past 224,000 years. *Nature* **451**, 1090–1093 (2008).
4. Kelly, M. J. *et al.* High resolution characterization of the Asian Monsoon between 146,000 and 99,000 years B.P. from Dongge Cave, China and global correlation of events surrounding Termination II. *Palaeogeogr. Palaeoclimatol. Palaeoecol.* **236**, 20–38 (2006).
5. Yoshimura, K., Kanamitsu, M., Noone, D. & Oki, T. Historical isotope simulation using Reanalysis atmospheric data. *J. Geophys. Res.* **113**, 2008JD010074 (2008).
6. Hercman, H. & Pawlak, J. MOD-AGE: An age-depth model construction algorithm. *Quat. Geochronol.* **12**, 1–10 (2012).
7. Cheng, H. *et al.* Ice Age Terminations. *Science* **326**, 248–252 (2009).
8. Uechi, Y. & Uemura, R. Dominant influence of the humidity in the moisture source region on the ^{17}O-excess in precipitation on a subtropical island. *Earth Planet. Sci. Lett.* **513**, 20–28 (2019).
9. He, S. *et al.* Understanding tropical convection through triple oxygen isotopes of precipitation from the maritime continent. *JGR Atm.* **126**, e2020JD033418 (2021).
10. Sha, L. *et al.* A novel application of triple oxygen isotope ratios of speleothems. *Geochim. Cosmochim. Ac.* **270**, 360–378 (2020).
11. Mokeddem, Z. & McManus, J. F. Persistent climatic and oceanographic oscillations in the subpolar North Atlantic during the MIS 6 glaciation and MIS 5 interglacial. *Paleoceanography* **31**, 758–778 (2016).
12. Tzedakis, P. C. *et al.* Enhanced climate instability in the North Atlantic and southern Europe during the Last Interglacial. *Nat. Commun.* **9**, 4235 (2018).
13. Irvalı, N. *et al.* Evidence for regional cooling, frontal advances, and East Greenland Ice Sheet changes during the demise of the last interglacial. *Quat. Sci. Rev.* **150**, 184–199 (2016).
14. Lisiecki, L. E. & Stern, J. V. Regional and global benthic δ^{18}O stacks for the last glacial cycle. *Paleoceanography* **31**, 1368–1394 (2016).
15. Barker, S. et al. Icebergs not the trigger for North Atlantic cold events. *Nature* **520**, 333–336 (2015).
16. Böhm, E. *et al.* Strong and deep Atlantic meridional overturning circulation during the last glacial cycle. *Nature* **517**, 73–76 (2015).
17. Deaney, E. L., Barker, S. & Van De Flierdt, T. Timing and nature of AMOC recovery across Termination 2 and magnitude of deglacial CO_2 change. *Nat. Commun.* **8**, 14595 (2017).
18. Martrat, B., Jimenez-Amat, P., Zahn, R. & Grimalt, J. O. Similarities and dissimilarities between the last two deglaciations and interglaciations in the North Atlantic region. *Quat. Sci. Rev.* **99**, 122–134 (2014).

19. Stoll, H. M. *et al.* Rapid northern hemisphere ice sheet melting during the penultimate deglaciation. *Nat. Commun.* **13**, 3819 (2022).
20. Bereiter, B. *et al.* Revision of the EPICA Dome C CO_2 record from 800 to 600 kyr before present: Analytical bias in the EDC CO_2 record. *Geophys. Res. Lett.* **42**, 542–549 (2015).
21. Li, M., Hinnov, L. & Kump, L. Acycle: Time-series analysis software for paleoclimate research and education. *Comput. & Geosci.* **127**, 12–22 (2019).
22. Jaffey, A. H., Flynn, K. F., Glendenin, L. E., Bentley, W. C. & Essling, A. M. Precision measurement of half-lives and specific activities of ^{235}U and ^{238}U. *Phys. Rev. C* **4**, 1889–1906 (1971).
23. Cheng, H. *et al.* Improvements in ^{230}Th dating, ^{230}Th and ^{234}U half-life values, and U–Th isotopic measurements by multi-collector inductively coupled plasma mass spectrometry. *Earth Planet. Sci. Lett.* **371–372**, 82–91 (2013).
