# Supplementary material for: Asian summer monsoon variability across Termination II and implications for ice age terminations
Source: Nat Commun. 2025 May 30;16:5025. doi: 10.1038/s41467-025-60398-w (PMC12125170; doi:10.1038/s41467-025-60398-w)
Supplement: Supplementary file 3 — Source Data [file 41467_2025_60398_MOESM3_ESM.zip › Source Data/Source data and code for Supplementary Figure 5/readme.html]

***Supplementary Material Code***

The supplementary material folder contains the file structure necessary to run code in Matlab and plot SI Figure S5. The main folder contains the code “ChineseCavesPlotter.m” and output plots and files.

 

**Folder “data”**

Data used to make SI Figure S5, including new data from this study:

- “tradData\_cc\_ChineseCaves.xlsx” contains the traditional stable isotope data
- “tripleOdata\_ChineseCaves\_V03” contains raw and normalized triple oxygen data for samples and standards

 

**Folder “functions”**

Functions for SI Figure S5

- “calciteToWater.m” models isotope fractionation between calcite and water
- “formT\_MC\_func.m” assesses the temperature sensitivity of the trend of reconstructed formation waters
- “ols.m” performs ordinary least squares regression
- “panEvapFunc.m” models pan evaporation
